# Subchronic Infection of* Porphyromonas gingivalis* and* Tannerella forsythia* Stimulates an Immune Response but Not Arthritis in Experimental Murine Model

**DOI:** 10.1155/2017/2052938

**Published:** 2017-06-06

**Authors:** Jorday Hernández-Aguas, José Luis Montiel-Hernández, Myriam A. De La Garza-Ramos, Rosa Velia Ruiz-Ramos, Erandi Escamilla García, Mario Alberto Guzmán-García, Esperanza Raquel Ayón-Haro, Mario Alberto Garza-Elizondo

**Affiliations:** ^1^Universidad Autónoma de Nuevo Leon, Centro de Investigación y Desarrollo en Ciencias de la Salud (CIDICS), Facultad de Odontología, Calle Dr. Eduardo Aguirre Pequeño s/n, Colonia Mitras Centro, 64460 Monterrey, NL, Mexico; ^2^Universidad Autónoma del Estado de Morelos, Facultad de Farmacia, Avenida Universidad 1001, 62209 Cuernavaca, MOR, Mexico; ^3^Universidad Autónoma de Nuevo Leon, Facultad de Medicina Veterinaria y Zootecnia, Centro de Investigación y Desarrollo en Ciencias de la Salud (CIDICS), Calle Francisco Villa s/n Colonia Ex-Hacienda el Canada, 66050 Escobedo, NL, Mexico; ^4^Universidad de San Martín de Porres, Laboratorio de Investigación en Biología Oral y Molecular, Ciudad Universitaria, Jr. Las Calandrias N° 151-291, Santa Anita, Lima 15009, Peru

## Abstract

Studies have proposed that* Porphyromonas gingivalis (Pg)* and* Tannerella forsythia (Tf)* promote a nonspecific inflammatory response that could produce systemic disease. Oral inoculation of* Pg* and* Tf* on the immune and arthritis response was evaluated in BALB/C mice divided into four groups: (1) sham; (2) food contaminated with* Pg/Tf*; (3) complete Freund's adjuvant (CFA) +* Pg/Tf*; and (4) CFA alone. CFA was administered subcutaneously on days 1 and 14. The arthritis response was monitored for 21 days after day 14 of CFA administration. IL-1*β* and IL-6 were determined in serum. T cell activation was evaluated by CD25 in salivary lymph nodes or mouse spleen. Pad inflammation appeared by day 19 in the CFA group, but animals with bacteria inoculation presented a delay. A significant increase in IL-6 was found in Groups 3 and 4, but not with respect to IL-1*β*. We observed an increase in CD25 in cells derived from cervical nodes and in animals with bacteria inoculation and CFA. A local immune response was observed in mice inoculated with* Pg* and* Tf* (T cell activation); a systemic response was observed with CFA. Since pad inflammation was delayed by bacterial inoculation this suggests that local T cell activation could decrease pad inflammation.

## 1. Introduction

Rheumatoid arthritis (RA) is an autoimmune joint disease that results in painful joint deformity and immobility [1]. It is characterized by symmetrical polyarticular inflammation of small and large joints with systemic involvement. Its annual incidence worldwide is about 40/100,000 with a lifetime risk of RA in women of 3.6%. The disease is also more frequent in women with a ratio of 2 : 1 to 3 : 1 [2].

The oral microbiota includes pathogens species that normally cause infections limited to the oral cavity; however, when these microorganisms or their components enter the circulation or connective tissues, they can activate a local or systemic immune response. In particular, chronic alterations in the sulcus microbiota balance could induce a very common inflammatory disease called periodontitis which suggests an unbalance between beneficial and pathogenic oral bacteria that induce inflammation and structural destruction of the support system of the tooth [3]. In recent years, several studies suggest that periodontitis could favor systemic diseases, such as RA [4–6]. In this regard, some experimental studies in animal models have confirmed that oral infection with* P. gingivalis* or* T. forsythia* is capable of activating an immune response and modulating arthritis development [7, 8]; however, the inducing mechanisms remain obscure.

Several basic and clinical studies have proposed that the periodontopathogenic bacteria* P. gingivalis *can secrete an enzyme with peptidyl arginine deiminase activity that could modify host peptides and proteins favoring, in theory, an autoimmune disorder [9]. Similarly, other posttranslational protein modifications such as carbamylation could favor alterations in the immune response and potentially explain the development of systemic disorders [10]. In this respect, it is critical to understand the routes by which the oral microenvironment could exert an influence on the local immune response as a strategy that explains subsequent systemic disorders. Our goal in this study was to evaluate in a subchronic murine model if bacteria inoculation alone was enough to induce immunological effects or modulate experimental arthritis inflammation.

## 2. Materials and Methods

Male BALB/C mice, 8–12 weeks of age, were used for the development of experimental arthritis. Four experimental groups of three mice each were formed: (1) sham; (2) inoculation with* P. gingivalis (Pg)/T. forsythia (Tf)*; (3) complete Freund's adjuvant + inoculation with* Pg/Tf*; and (4) Freund's complete adjuvant alone.

Trypticase soy broth supplemented with hemin and vitamin k was prepared for culturing* P. gingivalis* and* T. forsythia*. Bacteria were first activated in an anaerobic chamber. After activation, bacteria were cultured at 37°C for 24 hrs [11]. Subsequently, the bacteria were inoculated into the food by immersion in growth culture. The food mixture was allowed to dry for at least 2 days in an anaerobic chamber. Clean and contaminated food were distributed in 50 g aliquots, which were changed at the beginning of each week. On day 14, 10 *μ*L of contaminated culture medium was inoculated by pipette into the oral cavities of the two groups that would receive 50 *μ*L of contaminated food (Group 2* [Pg/Tf]* and Group 3 [CFA +* Pg/Tf*] every other day until the end of the study). Bacterial density was 0.5 McFarland standard as defined in the CLSI standard [12].

To induce experimental arthritis, two subcutaneous injections of 50 *μ*L of complete Freund's adjuvant (CFA) [*Mycobacterium tuberculosis* extract, 1 mg/mL] (Sigma-Aldrich, St. Louis, MO) were applied at the base of the tail of the mice in Groups 3 and 4 on days 1 and 14 [13].

Evaluation of the inflammatory events was performed with a vernier every third day for thirty-five days. The extent of swelling of the hind limbs was measured from the mouse heel to the base of the phalanges; thickness was measured in mm from the anterior to the posterior part of the paw (Table 1). Movement monitoring was classified according to an arbitrary system comprising 4 different levels, expressing results as an arithmetic mean. Additionally, the body weight of the animals was monitored every two to three days.

On days 14 and 35, saliva samples were obtained with sterile swabs to confirm the presence of the inoculated bacteria. Confirmation was achieved by observation in a Neubauer chamber. First, the swabs of each group were placed in Eppendorf tubes with trypticase soy to perform the bacterial count. These samples were left at 37°C for 24 hrs. After 24 hours, 1 *μ*L of the suspension was placed on a slide using a pipette, filling the counting chamber by capillarity; bacterial cells were counted in one of the larger 25 frames. Gram-staining was performed to demonstrate the presence of bacteria. Real-time qPCR was performed to determine bacterial viability. Virulence genes controlled by the luxS gene in* P. gingivalis* were measured to confirm viability and their natural condition since these cultures can modify their virulence expression patterns. This is because microbiological handling is complicated and can affect growth efficiency in culture media [14]. For this experiment the following primers were used: For* P. gingivalis*, CGGAGCCGGAAAGAAGG (forward), GCAGCACCCACGTAAAGAACAG (reverse), for* T. forsythia,* GCGTATGTAACCTGCCCGCA (forward), GAAGGCAGCTTACTAAGG (reverse), and as a control, GAPDH, TTGGAACTGGAACACGTTGTG (forward) and TAAAGCTATTGGTCTTGTTCCTG (reverse).

At the end of 35 days, the mice were sacrificed. Serum samples were obtained by intracardiac puncture and after clotting, the serum was recovered by centrifugation. Samples were kept at 4°C until analysis on the following day. Additionally, spleen and cervical nodes were recovered from each group and processed to obtain pooled cell samples. ELISA was performed to determine IL-1*β* and IL-6 levels (Peprotech Mexico S.A de C.V., Mexico City, Mexico). To evaluate expression of the activation marker CD25 in spleen and lymph node lymphocytic cells, tissues were isolated after sacrifice of the mice; cells were separated from tissue and fixed with 1% paraformaldehyde. The following day, these cells were incubated with FITC-coupled antibody anti-CD25 (Pharmagen, Lahore, Pakistan). Analysis was done on a FACscalibur flow cytometer.

## 3. Results

### 3.1. Arthritis Model

**Figure 1:**
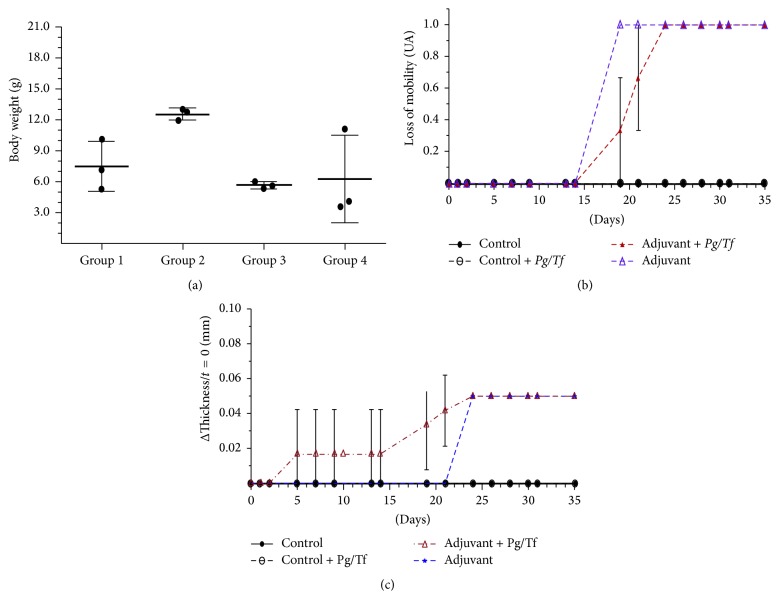
(a) Effect of treatment with CFA and bacterial inoculation on change in body weight of experimental animals. The results of 3 animals per group, the mean and standard deviation are shown. To determine the change in body weight of each animal, the difference was obtained between the weight at the start of the experiment (day 0) and at the end of the experiment (day 35). The evaluation was performed based on a dichotomous criterion: normal mobility and decreased mobility. With the Kruskal-Wallis test, the difference between the groups did not reach statistical significance (*p* = 0.0784), although the change in body weight of Group 2 seemed to be greater in relation to the other groups. (b) Effect of treatment with CFA and bacterial inoculation on loss of mobility. Results of 3 animals per group; the mean and standard deviation are shown. To determine the change of mobility of each animal, each evaluation was performed based on a dichotomous criterion: normal mobility and reduced mobility. Solid black line, sham (Group 1); black dashed line, sham +* Pg/Tf* (Group 2); blue dashed line, CFA +* Pg/Tf* (Group 3); red dashed line, CFA alone (Group 4). A loss of mobility was observed from day 19 in the CFA group, while the loss of mobility was complete in the CFA +* Pg/Tf* group starting on day 24 of the study. (c) Effect of treatment with CFA and bacterial inoculation on hind leg thickness. Results correspond to the mean and standard deviation of the two hind limbs of 3 animals per group. The change in thickness (ΔThickness) was determined based on the difference between the initial thickness of the foot pad and thickness during the different study times. Solid black line, sham (Group 1); black dashed line, sham +* Pg/Tf* (Group 2); blue dashed line, CFA +* Pg/Tf* (Group 3); red dashed line, CFA alone (Group 4). The group treated with CFA without* Pg/Tf* showed an increase in thickness after day 19. The group treated with CFA and with* Pg/Tf* showed a delayed increase (day 24).

The mobility of the animals and body weight in the 4 groups were monitored as indirect markers of inflammation of CFA treatment. As seen in Figure 1(a), statistically, body weight did not differ between Groups 1 (7.49 ± 2.42 g), 3 (5.64 ± 0.35 g), and 4 (6.26 ± 4.2 g). In contrast, the group inoculated only with* Pg/Tf* showed a greater increase in body weight (12.56 ± 0.56 g). Otherwise, the mobility of animals was significantly different between groups (Figure 1(b)), showing that groups treated with CFA lose mobility just after the second immune challenge (day 19) until the end of the experiment (day 35). Interestingly, the group receiving oral treatment with bacteria showed a significant delay in the loss of displacement potentially suggesting a minor inflammatory status.

The hind legs of the experimental animals were also evaluated based on the change in thickness. Neither the sham group nor the group inoculated with* Pg/Tf* showed any variation in hind leg thickness. The group of animals treated with CFA showed no statistically significant increase up to day 5 of treatment, but after day 19, the increase was statistically significant. This change coincides with the effect in response to the second application of CFA (day 14) (Figure 1(c)). Finally, the CFA group inoculated with* Pg/Tf* showed no increase in foot pad thickness during the first 19 days of the experiment; however, by day 24, all showed a significant increase, similar to the effect of the group treated only with CFA. This seems to suggest that bacterial inoculation somehow decreases the propensity for moderate inflammation (window between 5–22 days) and delays the onset of the increase in thickness (day 19 to 24).

### 3.2. Immunological Effects of Oral Bacteria

**Figure 2:**
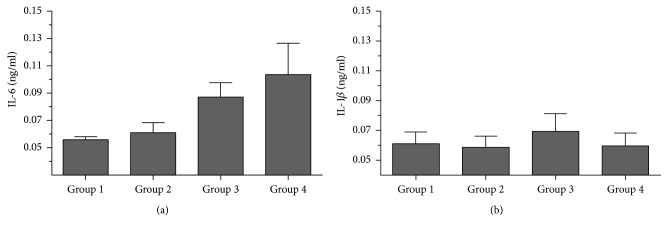
(a) Effect of the inoculation of periodontopathogenic bacteria on serum IL-6 levels. The values shown are the mean of three mice analyzed by study group plus their respective mean. Group 1: untreated sham; Group 2: mice inoculated with* Pg/Tf*; Group 3: inoculated intradermally with CFA +* Pg/Tf*; and Group 4: CFA inoculated intradermally. These values show a significant increase in IL-6 levels of Group 1 versus Group 2, although a significant difference was not demonstrated (*p* = 0.128) in contrast with Group 3 (*p* = 0.0262). A significant difference was found in Group 1 versus Group 4 (*p* = 0.0555). (b) Effect of the inoculation of periodontopathogenic bacteria on serum levels of IL-1*β*. Murine blood was drawn on day 35 after euthanasia and was centrifuged and analyzed the next day. IL-1*β* in the supernatant was determined by ELISA. Groups: 1, 2, 3, 4. Bars represent the mean ± standard deviation of three replicates from three animals per group; these did not show statistically significant increases. PRISM v.6.0 was used for data analysis.

In order to evaluate the effect of oral inoculation of bacteria strains on the immune response, plasma levels of two inflammatory cytokines, IL-1*β* and IL-6, were determined. As shown in Figure 2(a), IL-6 levels were statistically similar between Groups 1 (sham) and 2 (bacterial inoculation). Group 4 (CFA) showed a significant increase in IL-6 levels compared to the sham or bacterial inoculation groups (*p* < 0.05), despite the fact that it was the experimental group where there was greater variation in the results. The group of mice treated with CFA and bacterial inoculation also showed increased IL-6 levels with a significantly higher mean than that observed in the sham group (*p* = 0.0262). It is noteworthy that the mean concentration of serum IL-6 levels was lower in the case of animals in Group 3 compared to Group 4, although no statistical significance was reached.

IL-1*β* serum levels were not significantly altered between the different experimental groups, showing a variation between 47 and 82 pg/mL (Figure 2(b)). Although a slight increase in cytokine levels in Group 3 (CFA +* Pf/Tf*) was observed, the standard deviation of the different groups cancels any proposed difference. Additional statistical analyses by the Kruskal-Wallis test showed no significant differences in IL-1*β* levels due to the effect of CFA or bacterial inoculation.

**Figure 3:**
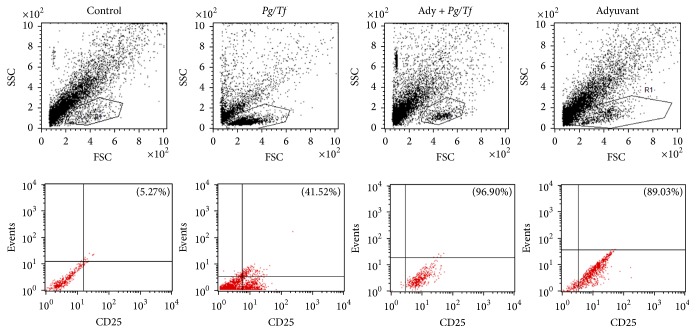
Flow cytometry of the frequency of regulatory T cells (CD25+) induced by* Pg/Tf*, CFA in BALB/c mice.

In order to evaluate possible effects on the activation of immune system cells, lymphoid cells derived from cervical nodes and the spleens of animals were analyzed according to the percentage and relative surface presence of the activation marker CD25 (Figure 3). Evaluation of splenocyte preparations resulted in similar CD25 levels in comparison to sham animals, suggesting that oral inoculation of* P. gingivalis* and* T. forsythia* during 35 days was not enough to induce systemic activation of T cells (data not shown). Otherwise, in lymphoid cells derived from cervical nodes, we clearly observed an activation effect. In comparison to the sham group, animals inoculated only with bacteria (Group 2) showed an eightfold increase of CD25 levels (5.27 versus 41.52%). However, animals treated only with CFA in the absence or presence of oral bacteria showed a higher percentage of CD25-positive cells (89.03% and 96.90%, resp.), confirming that a complete-adjuvant challenge exerts strong immune system stimulation. Although this was not significantly different, animals treated with CFA and bacteria inoculation showed the highest levels of CD25, suggesting that oral bacteria could exert additional stimulation. It is noteworthy that only oral inoculation of* P. gingivalis* and* T. forsythia* was enough to stimulate local but not systemic lymphocytes, suggesting that oral microbiota disorders can potentially alter the immune response, even if systemic symptoms are not observed.

## 4. Discussion

Periodontal colonization of pathogenic bacteria (*P. gingivalis* and* T. forsythia*) clearly showed pathogenic and inflammatory effects on the host oral microenvironment [15, 16] but its consequences on the systemic host immune response remains poorly studied. In this regard, some studies have shown that periodontitis can induce platelet aggregation and activation [17], together with an increase in the levels of inflammatory cytokines such as IL-6 and TNFa [18, 19]. Additionally, some studies have shown, as a response of periodontitis, chemotaxis and activation of neutrophils or monocytes [19, 20], suggesting a very dynamic and modulated communication between the oral microenvironment and the immune system [21]. Additionally, these results come to support the hypothesis that proposes that chronic oral disease could favor systemic inflammatory disorders such as rheumatoid arthritis [22, 23].

In order to characterize the biological mechanisms employed by the oral microenvironment in the host immune response, we proposed to evaluate their effect in a subchronic CFA-induced arthritis model. First, CFA induced an increase in footpad thickness which caused a decrease in mobility with and without inoculation of* P. gingivalis* and* T. forsythia* (Figure 1). Additionally, increased serum levels of IL-6, but not of IL-1*β*, were observed. Although slightly lower levels were observed, the group treated with CFA and inoculated with the mixture of bacteria also showed increased IL-6 levels. Bacterial inoculation, without treatment with CFA, resulted in a significant increase in the percentage of CD25^+^ cells derived from cervical lymph nodes, but not from spleen-derived cells. On the other hand, treatment with CFA also caused a significant increase in the percentage of CD25^+^ cells in Groups 3 (97%) and 4 (86%). However, it did not alter systemic immunological variables (spleen). The greatest inflammatory (or in less time) effect in mice was found in Group 4. This could suggest that inoculation of periodontopathogenic bacteria modulates the inflammatory response in this experimental murine model. This is similar to the results of Bartold et al. [5] who observed established chronic inflammation with increased limb length. In our study, the hind limbs showed an increase in diameter one week after the second immunization with CFA. Also, in a previous study by Barajas-Torres [4], inflammation of the anterior and posterior joints was produced in mice immunized with the thymic hormonal factor, tactivin, compared to controls. Different studies state the need to increase the observation time after immunization or increase the number of applications with CFA with the aim of determining the inflammatory response [6, 20, 24].

Additionally, we observed a higher prevalence of arthritis in the group inoculated with* Pg/Tf* and in the group with* Pg/Tf* plus CFA, similar to the research by Kinloch et al. [25] who found increased ankle thickness in mice after a second inoculation with CFA.

Although we observed a significant increase in IL-6 levels in the course of the experiment, it may be necessary to increase the length of time and number of immunizations or the application of a new inflammatory inductor to find changes in IL-*β* levels. Since several studies have confirmed the critical role played by IL-1*β* in the innate immune response [26], it is not clear why we did not observe alterations due to the application of CFA or if there was an affect caused by bacterial inoculation.

On the other hand, it is unclear whether or not chronic oral infection with* P. gingivalis* should occur prior to CFA application. In this respect, employing a chronic murine model of* P. gingivalis *infection prior to arthritis induction, Marchesan et al. described an increased activation of the immune system that favors regulatory T cell responses, which ultimately would accelerate the development of arthritis [27]. This suggests that chronic oral infection can influence the development of RA primarily through activation of pathways related to Th17.

Other studies suggest that, to observe a chronic periodontal disease and an inflammatory disease, a time range, a different strain immunized with another inflammatory inducer, the creation of a chronic extrasynovial lesion before immunization [5], an acid phosphatase test, and an analysis of a computed microtomography of the hind limb [7] are required.

Our current findings provide critical insight into autoimmune function and the number of samples in our study is statistically similar to the study of Falsetta et al. [28] and Klein et al. [29] who used different study conditions. This observation highlights the fundamental pathogenic role of the* P. gingivalis*/*T. forsythia* mixture and inflammatory adjuvants as a logical objective for increasing immunization and oral inoculations to obtain high levels of these inflammation modulators.

## Figures and Tables

**Table 1:** Inflammatory response of the hind limbs of Balb/c mice.

Groups	Hind limb swelling (mm)	Hind limb thickness (mm)
*Sham group+*		
Sham+	R: 1.4-L: 1.4	R: 2-L: 2
Sham+	R: 1.4-L: 1.4	R: 2-L: 2
Sham+	R: 1.4-L: 1.4	R: 2-L: 2
*Group 2 sham− *	R: 1.4-L: 1.4	R: 2-L: 2
*P. gingivalis* plus *T. forsythia*	R: 1.4-L: 1.4	R: 2-L: 2
*P.gingivalis.* plus *T. forsythia*	R: 1.4-L: 1.4	R: 2-L: 2
*Group 3*	R: 1.4-L: 1.4	R: 2.5-L: 2.5
*P. gingivalis* plus Freud adjuvant	R: 1.4-L: 1.4	R: 2.5-L: 2.5
*P. gingivalis* plus Freud adjuvant	R: 1.4-L: 1.4	R: 2.5-L: 2.5
*Group 4*	R: 1.4-L: 1.4	R: 2.5-L: 2.5
Freud adjuvant	R: 1.5-L: 1.5	R: 2.5-L: 2.5
Freud adjuvant	R: 1.4-L: 1.4	R: 2.5-L: 2.5

R = right; L = left.
