# Corrigendum to “Subchronic Infection of *Porphyromonas gingivalis* and *Tannerella forsythia* Stimulates an Immune Response but Not Arthritis in Experimental Murine Model”

**DOI:** 10.1155/2018/6093609

**Published:** 2018-10-01

**Authors:** Jorday Hernández-Aguas, José Luis Montiel-Hernández, Myriam A. De La Garza-Ramos, Rosa Velia Ruiz-Ramos, Erandi Escamilla García, Mario Alberto Guzmán-García, Esperanza Raquel Ayón-Haro, Mario Alberto Garza-Elizondo

**Affiliations:** ^1^Universidad Autónoma de Nuevo Leon, Centro de Investigación y Desarrollo en Ciencias de la Salud (CIDICS), Facultad de Odontología, Calle Dr. Eduardo Aguirre Pequeño s/n, Colonia Mitras Centro, 64460 Monterrey, NL, Mexico; ^2^Universidad Autónoma del Estado de Morelos, Facultad de Farmacia, Avenida Universidad 1001, 62209 Cuernavaca, MOR, Mexico; ^3^Universidad Autónoma de Nuevo Leon, Facultad de Medicina Veterinaria y Zootecnia, Centro de Investigación y Desarrollo en Ciencias de la Salud (CIDICS), Calle Francisco Villa s/n Colonia Ex-Hacienda el Canada, 66050 Escobedo, NL, Mexico; ^4^Universidad de San Martín de Porres, Laboratorio de Investigación en Biología Oral y Molecular, Ciudad Universitaria, Jr. Las Calandrias N° 151-291, Santa Anita, Lima 15009, Peru

In the article titled “Subchronic Infection of *Porphyromonas gingivalis* and *Tannerella forsythia* Stimulates an Immune Response but Not Arthritis in Experimental Murine Model” [1], there was an error in Table 1 where “Freud's adjuvant” should be changed to “Freund's adjuvant.” The formatting of the table is also being corrected for clarity. The correct table is as follows.

## Figures and Tables

**Table 1:** Inflammatory response of the hind limbs of Balb/c mice.

Groups	Hind limb swelling (mm)	Hind limb thickness (mm)
*Group 1*		
Sham	R: 1.4-L: 1.4	R: 2-L: 2
Sham	R: 1.4-L: 1.4	R: 2-L: 2
Sham	R: 1.4-L: 1.4	R: 2-L: 2

*Group 2*		
Sham + *P. gingivalis* plus *T. forsythia*	R: 1.4-L: 1.4	R: 2-L: 2
Sham + *P. gingivalis* plus *T. forsythia*	R: 1.4-L: 1.4	R: 2-L: 2
Sham + *P. gingivalis* plus *T. forsythia*	R: 1.4-L: 1.4	R: 2-L: 2

*Group 3*		
*Pg/Tf* plus Freund's adjuvant	R: 1.4-L: 1.4	R: 2.5-L: 2.5
*Pg/Tf* plus Freund's adjuvant	R: 1.4-L: 1.4	R: 2.5-L: 2.5
*Pg/Tf* plus Freund's adjuvant	R: 1.4-L: 1.4	R: 2.5-L: 2.5

*Group 4*		
Freund's adjuvant	R: 1.4-L: 1.4	R: 2.5-L: 2.5
Freund's adjuvant	R: 1.5-L: 1.5	R: 2.5-L: 2.5
Freund's adjuvant	R: 1.4-L: 1.4	R: 2.5-L: 2.5

R = right; L = left; *Pg/Tf* = *P. gingivalis* plus *T. forsythia*; sham = placebo.
